# Single-Target Regulators Constitute the Minority Group of Transcription Factors in *Escherichia coli* K-12

**DOI:** 10.3389/fmicb.2021.697803

**Published:** 2021-06-18

**Authors:** Tomohiro Shimada, Hiroshi Ogasawara, Ikki Kobayashi, Naoki Kobayashi, Akira Ishihama

**Affiliations:** ^1^School of Agriculture, Meiji University, Kawasaki, Japan; ^2^Research Center for Supports to Advanced Science, Division of Gene Research, Shinshu University, Nagano, Japan; ^3^Research Center for Fungal and Microbial Dynamism, Shinshu University, Nagano, Japan; ^4^Department of Frontier Science, Hosei University, Koganei, Japan; ^5^Micro-Nano Technology Research Center, Hosei University, Koganei, Japan

**Keywords:** transcription regulator, single-target transcription factor, genomic SELEX, *Escherichia coli* K-12, QseA, RspR

## Abstract

The identification of regulatory targets of all transcription factors (TFs) is critical for understanding the entire network of genome regulation. A total of approximately 300 TFs exist in the model prokaryote *Escherichia coli* K-12, but the identification of whole sets of their direct targets is impossible with use of *in vivo* approaches. For this end, the most direct and quick approach is to identify the TF-binding sites *in vitro* on the genome. We then developed and utilized the gSELEX screening system *in vitro* for identification of more than 150 *E. coli* TF-binding sites along the *E. coli* genome. Based on the number of predicted regulatory targets, we classified *E. coli* K-12 TFs into four groups, altogether forming a hierarchy ranging from a single-target TF (ST-TF) to local TFs, global TFs, and nucleoid-associated TFs controlling as many as 1,000 targets. Using the collection of purified TFs and a library of genome DNA segments from a single and the same *E. coli* K-12, we identified here a total of 11 novel ST-TFs, CsqR, CusR, HprR, NorR, PepA, PutA, QseA, RspR, UvrY, ZraR, and YqhC. The regulation of single-target promoters was analyzed in details for the hitherto uncharacterized QseA and RspR. In most cases, the ST-TF gene and its regulatory target genes are adjacently located on the *E. coli* K-12 genome, implying their simultaneous transfer in the course of genome evolution. The newly identified 11 ST-TFs and the total of 13 hitherto identified altogether constitute the minority group of TFs in *E. coli* K-12.

## Introduction

The model prokaryote *Escherichia coli* K-12, one of the enterobacteria, inhabits virtually every environment on earth. Outside host animals, *E. coli* is directly exposed to stressful environment in nature. For adaptation and survival, *E. coli* constantly monitors physical, chemical and biological conditions in the environment, and modifies the expression pattern of its genome containing more than 4,500 genes. The major regulatory step of genome expression is transcription, which is carried out by a single species of RNA polymerase (RNAP). The model prokaryote *E. coli* K-12 contains about 2,000 molecules of RNAP core enzyme (Ishihama, 2010, 2012), which is less than the total number (approximately 4,500) of genes on its genome. The pattern of RNAP utilization between 4,500 genes is, however, modulated through interaction with two groups of the regulatory proteins, i.e., seven species of the sigma factor with promoter recognition activity in the first step (Ishihama, 2000; Gruber and Gross, 2003; Gourse et al., 2006) and approximately 300 species of DNA-binding transcription factors (TFs) in the second step (Pérez-Rueda and Collado-Vides, 2000; Babu and Teichmann, 2003; Ishihama et al., 2016). Based on the DNA-binding motifs, we classified these TFs into 63 families (Ishihama et al., 2016; also cited in the TEC database^1^). The activator-type TFs interact directly with one of the RNAP subunits for function (Ishihama, 1993, 2010; Busby and Ebright, 1999) whereas the repressor-type TFs interfere with transcription initiation by interfering with RNAP binding to the promoters (Cowell, 1994; Gralla, 1996). Some repressors bind upstream of the promoters and interfere with transcription initiation through protein-protein contact with promoter-bound RNAP, thereby preventing promoter escape (Yamamoto and Ishihama, 2003). Some repressors function as road-block through protein-protein interaction between TF and RNAP. The binding of TFs near the promoter leads to an increase in their local concentration regulates, thereby enabling effective protein-protein interactions between RNAP and TFs for modulation of the promoter selectivity of transcriptase. For modeling the regulatory networks of genome transcription involving all seven sigma factors and all 300 TFs, the identification of the association between each of these regulatory proteins and their direct targets is a major bottleneck.

Advanced genome-wide research technologies such as transcriptomics (Richmond et al., 1999; Oshima et al., 2002; Grainger et al., 2005), ChIP-chip (Bulyk, 1999; Grainger et al., 2009), and ChIP-seq analyses (Kahramanoglou et al., 2010; Antipov et al., 2017) have been widely employed to identify transcription patterns of the genome *in vivo* in the presence and absence of a test regulator or after over-expression of the test regulator. Mainly based on these *in vivo* data, approximately 70–80% of the estimated 300 TFs in *E. coli* have been linked to at least one regulatory target gene or operon in the genome as listed in databases such as such as EcoCyc (Keseler et al., 2005, 2011) and Regulon DB (Salgado et al., 2006, 2013; Gama-Castro et al., 2016). From the *in vivo* data alone, it is difficult to discriminate the direct and indirect targets. Furthermore, it is in principle difficult to identify the whole set of direct regulatory targets *in vivo* because the binding of TFs to their target DNA is interfered by more than 500 species of co-existing DNA-binding proteins in *E. coli* cells, including 300 TFs (Ishihama, 2012; Ishihama et al., 2016) and more than 200 species of other DNA-binding proteins involved in DNA functions (Ishihama, 2009). In addition, another issue with using unselected data sets of *in vivo* transcription is related to the difference in genetic backgrounds of bacterial strains used in experiments performed in different laboratories. Recently it turned clear that large amounts of sequence difference exist in the genome between different *E. coli* strains (Land et al., 2015; Dunne et al., 2017). For instance, the difference between seven sigma factors are observed not only between different *E. coli* strains but also between different laboratory stocks of the same *E. coli* strain (Jishage and Ishihama, 1997; Ishihama, 2009). In addition, some of the regulatory targets listed in EcoCyc and Regulon databases were predicted *in silico* based on the presence of known TF recognition sequence with different levels of accuracy but without experimental confirmation.

To overcome the problems encountered in the *in vivo* approaches as noted above, we decided to employ an *in vitro* approach. As bacterial TFs generally bind to the recognition sequences located near the promoters of regulatory target gene or operons, we developed the genomic SELEX (systematic evolution of ligands by exponential enrichment; hereafter referred to as gSELEX) as a shortcut approach for the identification of regulatory targets under the direct control of TFs (Shimada et al., 2005, 2018a; Ishihama et al., 2016). For identification of the whole set of direct regulatory targets for each TF, gSELEX offers a number of advantages over *in vivo* analyses (Shimada et al., 2018a). First, the TF-binding site can be identified in the absence of other DNA-binding proteins. Second, the TF-binding affinity to targets can be monitored by changing the TF-DNA probe mixing ratio or by controlling the SELEX cycles. Third, the possible influence of effectors and protein covalent modifications on the TF function can be easily examined (Shimada et al., 2014a). Fourth, only the direct targets of the test TF can be identified; the indirectly affected targets associated with *in vivo* data can be excluded (Shimada et al., 2018a). Noteworthy is that the TF proteins and DNA probes used in gSELEX screening were obtained from a single and the same *E. coli* K-12 strain, thereby eliminating the problems arising from the differences in genetic backgrounds. In the *in vitro* gSELEX screening, it is necessary to prepare purified TFs in functional forms. For some TFs, as yet unidentified effector ligands are needed for TF activation. Some TFs require other collaborator proteins are needed for function, together forming heterooligomers.

Using the gSELEX system, we have so far identified a complete set of constitutive promoters for five sigma factors (RpoD, RpoS, RpoH, RpoF, and RpoE) in *E. coli* K-12 W3110 (Shimada et al., 2014b, 2017). In parallel, a systematic gSELEX screening is in progress for the identification of the entire set of regulatory targets of all 300 DNA-binding TFs found in *E. coli* K-12 W3110, including both characterized and uncharacterized TFs. gSELEX screening is in particular useful for identification of regulatory targets of uncharacterized TFs with no known functions. The aim of gSELEX screening is to identify the whole set of promoters under the direct control of one specific test TF, and thus gSELEX is defined as a “TF-to-Promoter” approach. In parallel, we have also developed PS-TF (promoter-specific transcription-factor) screening system as a “Promoter-to-TF” approach for the detection of whole set of TFs involved in the regulation of one specific test promoter (Shimada et al., 2014c; Yoshida et al., 2018; Ogasawara et al., 2020).

**FIGURE 1:**
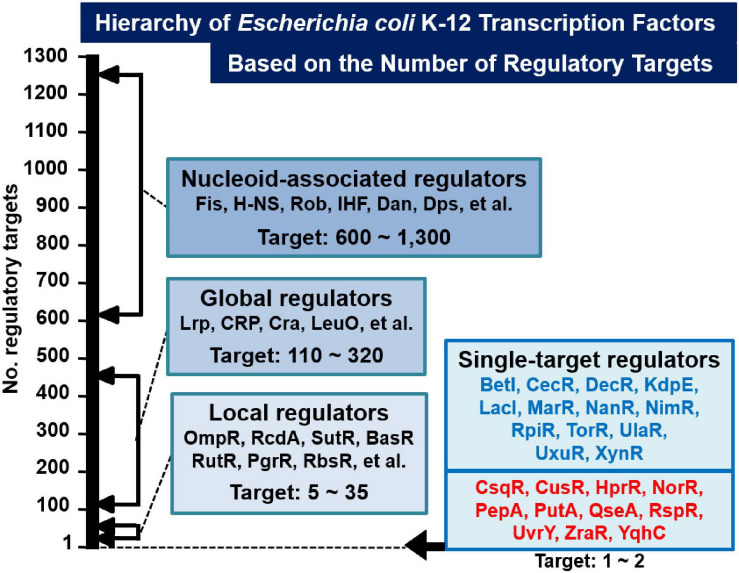
Classification of TFs of *Escherichia coli* K-12 W3110. Based on the number of regulatory targets identified by gSELEX screening, approximately half of a total of about 300 *E. coli* K-12 TFs were classified into four groups (36; see also TEC database www.shigen.nig.ac/ecoli/tec/). Some representative TFs are shown in each group. In the case of nucleoid-associated regulators, the indicated number represents the total number of TF-binding sites on the *E. coli* K-12 genome. The first version of the list of single-target TFs (ST-TFs) includes 13 TFs (Shimada et al., 2018b) (indicated in blue). In this report, we described the second version of the list, including 11 additional ST-TFs (indicated in red).

Based on the gSELEX screening results so far carried out for about 200 TFs, we have proposed a novel classification system of TFs: single-target regulators (number of targets, 1 to several), local TFs (targets ranging from 10 to 50), global regulators (more than 100 targets), and nucleoid-associated regulators (as many as 1,000 targets) (Figure 1). Once we get the list of regulatory targets for most of *E. coli* K-12 TFs, we will propose the detailed and improved classification system, in which the local regulators including ST-TFs will be grouped into a number of subgroup. Until that time, we classify the set of TFs regulating one to several targets will be classified into ST-TFs. Since the first molecular characterization of *E. coli* TF was performed for the single-target LacI (Lewis, 2005), LacI was recognized as a representative model TF system in *E. coli.* One unexpected finding of the gSELEX screening was the limited number (only less than 10% of total TFs) of LacI-type single-target TFs. Previously we reported a list of 13 single-target TFs (ST-TFs), including 9 known TFs (BetI, KdpE, LacI, MarR, NanR, RpiR, TorR, UlaR, and UxuR) and 4 uncharacterized TFs (YagI, YbaO, YbiH, and YeaM) (Shimada et al., 2018b). Since then we continued gSELEX screening for the rest of *E. coli* K-12 TFs and found additional ST-TFs, we decided to publish here the list of newly identified 11 ST-TFs, including CsqR, CusR, HprR, NorR, PepA, PutA, QseA, RspR, UvrY, ZraR, and YqhC. Regulatory functions are also analyzed in details for two hitherto uncharacterized TFs, QseA, and RspR.

## Materials and Methods

### *Escherichia coli* Strains and Culture Conditions

The genome of *E. coli* K-12 W3110 type-A (Jishage and Ishihama, 1997) was used as the source for construction of TF expression plasmids and DNA library used for SELEX screening of regulatory targets of TFs. *E. coli* BW25113 (Datsenko and Wanner, 2000) and its *qseA* and *rspR* knockout mutants (Baba et al., 2006) were obtained from the *E. coli* Stock Center (National Bio-Resource Center, NIG, Japan). *E. coli* DH5αwas used for amplification of plasmids. *E. coli* BL21 (DE3) was used for overexpression of all TFs. Cells were grown in LB medium with shaking at 37°C.

### Expression and Purification of TFs

All purified TFs used in this study were obtained from the purified *E. coli* TF collection of the Ishihama laboratory (Hosei University, Japan). In brief, the expression plasmid of all TFs was constructed according to the standard procedure (Yamamoto et al., 2005). The TF-coding sequences were purified via PCR using *E. coli* K-12 W3110 type-A genomic DNA as a template and were inserted into the pET21αvector. The His-tagged TFs were expressed in *E. coli* BL21 (DE3). His-tagged TFs were affinity-purified according to the standard procedure (Yamamoto et al., 2005). The purity of all the TFs used in this study was more than 95% as detected by staining of the PAGE gel.

### Genomic SELEX Screening of TF-Binding Sequences

Genomic SELEX (gSELEX) was performed according to the standard procedure (Shimada et al., 2005, 2018a) using each of the purified TFs and the collection of genomic DNA segments of *E. coli* K-12 W3110. The gSELEX described in this report was repeated three to six cycles depending on TF species. Mapping of final gSELEX fragments along the *E. coli* genome was performed by using a 43,450-feature DNA microarray (Oxford Gene Technology, United Kingdom). Since the summed length of 60 bp-long probe mixtures attached on the DNA array correspond to about 6.5-fold excess of the genome size, each TF-bound DNA should bind maximum 6 to 7 different probes, and thus approximately 300 bp-long TF-binding gSELEX segments should bind to more than six consecutive probes aligned at 105 bp intervals. This criterion was employed to check the accuracy of tilling array assay. The genomic SELEX sample was labeled with Cy3, while the reference SELEX sample was labeled with Cy5. After hybridization of both samples with the same DNA tilling array, Cy5/Cy3 ratio was measured for each probe. The scanned pattern was plotted along the *E. coli* K-12 genome. Non-specific peaks that appeared in the early stage of gSELEX disappeared after repeated cycles of gSELEX (Shimada et al., 2018a). All the SELEX-chip data described in this report were deposited in the “Transcription Factor Profiling of *Escherichia coli*” (TEC) database at the National Institute of Genetics (Ishihama et al., 2016; TEC, see text footnote 1).

### Gel Shift Assay

Gel shift assay was performed according to the standard procedure (Shimada et al., 2013). Probes containing the recognition target sequences of the test TFs (220 bp for *aaeX/qseA* spacer region and 500 bp for *rspA* promoter region) were generated by PCR amplification using a pair of primers (Supplementary Table 1A) and Ex Taq DNA polymerase (Takara, Kusatsu, Japan). For the assay, a mixture of each probe and purified TF was incubated at 37°C for 30 min in the gel shift buffer. After the addition of DNA loading solution, the mixture was directly subjected to PAGE (polyacrylamide gel electrophoresis). Probe DNA in gels was stained with GelRed (Biotium, Fremont, CA, United States) and was detected using LAS-4000 IR multi-color (GE Healthcare, Little Chalfont, United Kingdom).

### DNase I Footprinting Assay

DNase-I foot-printing assay was carried out under standard reaction conditions (Shimada et al., 2007). In brief, 1.0 pmol each of FITC-labeled probes and purified TF were mixed with the binding buffer; the mixture was incubated at 25°C for 30 min. After the incubation period, DNA was digested using 5 ng of DNase I (TaKaRa). After 30 s of digestion at 25°C, the reaction was terminated by adding 25 μL of phenol to the reaction mixture. DNA was precipitated from the aqueous layer using ethanol, dissolved in formamide dye solution, and electrophoresed on a 6% polyacrylamide gel containing 8 M urea with sequence ladder.

### Biofilm Assay

Biofilm formation was determined using the crystal violet staining method as described previously (Shimada et al., 2012). *E. coli* cells were grown in LB medium without NaCl at 28°C in a plastic tube. After 6 h of static cultivation, planktonic cells were discarded and the tube was washed twice with PBS (-). The cells attached to the tube were then stained with 0.1% crystal violet for 20 min at room temperature. After extensive washing with H_2_O, biofilm-bound crystal violet was extracted with 1 mL of 70% ethanol and measured for the density at OD_595__*nm*_ using plate reader MTP-880 (Corona).

### Northern Blot Analysis

Total RNA was extracted from *E. coli* cells in the exponential phase (OD_600_ = 0.3) or stationary phase (OD_600_ = 1.5) using ISOGEN solution (Nippon gene). RNA purity was confirmed by electrophoresis on a 1.5% agarose gel in the presence of formaldehyde, followed by staining with GelRed. Northern blot analysis was performed as described previously (Shimada et al., 2015a). DIG-labeled probes were prepared by PCR amplification using W3110 genomic DNA (50 ng) as template, a pair of primers (Supplementary Table 1B), DIG-11-dUTP (Roche), dNTPs as substrates, gene-specific forward and reverse primers, and Ex Taq DNA polymerase. Total RNA (3 μg) was incubated in formaldehyde-morpholinepropanesulfonic acid (MOPS) gel-loading buffer for 10 min at 65°C for denaturation, subjected to electrophoresis on a 1.5% agarose gel containing formaldehyde, and then transferred to a nylon membrane (Roche). Hybridization was performed on the DIG easy Hyb system (Roche) at 50°C overnight using a DIG-labeled probe. To detect the DIG-labeled probe, the membrane was treated with anti-DIG-AP Fab fragments and CDP-Star (Roche), and the resulting image was scanned with LuminoGraph I (Atto).

### Primer Extension Analysis

Primer extension analysis was performed according to the standard procedure using 5′-FITC-labeled probe (Umezawa et al., 2009), which was extended *in vitro* using AMV (avian myeloblastosis virus) reverse transcriptase (TaKaRa). *E. coli* K-12 was grown in LB medium at 37°C under aerobic conditions, and total RNA was extracted from exponentially growing cells (OD_600_ = 0.3). After incubation for 1 h at 50°C, DNA was extracted using phenol, precipitated using ethanol, and electrophoresed on a 6% polyacrylamide sequencing gel containing 7 M urea. Fluorescence-labeled DNA in gels was detected using the slab gel DNA sequencer DSQ-500L (Shimadzu).

### RT-qPCR Assay

RT-qPCR analysis was performed according to the standard procedure (Shimada et al., 2015b). *E. coli* cells were inoculated in LB medium at 37°C under aeration with constant shaking at 150 rpm until OD_600_ reached 0.3 or 1.5, following which total RNA was extracted. The total RNA was transcribed to cDNA using random primers and THUNDERBIRD SYBR qPCR RT set (TOYOBO, Osaka, Japan). Quantitative PCR (qPCR) was conducted using THUNDERBIRD SYBR qPCR mix (TOYOBO) and was performed using the LightCycler 480 system (Roche). The pairs of primers used in the experiment are described in Supplementary Table 1C. The cDNA templates were serially diluted fourfold and used in the qPCR assays. The qPCR mixtures, each containing 10 μL of THUNDERBIRD SYBR qPCR mix (TOYOBO), 1 μL of each primer (5 μM stock), 7 μL of water, and 1 μL of cDNA, were amplified under the following thermal cycling conditions: 95°C treatment for 2 min; 45 cycles of 10 s at 95°C and 20 s at 55°C; and incubation for 20 s at 72°C. The expression levels of 16S rRNA were used to normalize the RNA levels of test samples, and the relative expression levels were quantified using Relative Quantification software provided by Roche. The results presented are the averages of the results from three experiments.

## Results

### Identification of Novel Type-A Single-Target TFs

Most of the TF genes in the *E. coli* genome are located on the part of the *E. coli* genome that is closely connected with or is adjacent to their regulatory target genes, forming a gene organization in which TF and its regulatory targets exist as an adjacent set, herein referred to as type-A gene organization. For instance, *lacI* and *lacZYA* genes form a typical type-A organization (Shimada et al., 2018b). Type-A gene organization brings about a selective benefit for efficient propagation and integration for genome evolution through horizontal gene transfer among the bacterial kingdom (Lawrence, 1997; Rubinstein et al., 2011). Previously, we had reported 13 ST-TFs (BetI, CecR, DecR, KdpE, LacI, MarR, NanR, NimR, RpiR, TorR, UlaR, UxuR, and XylR) (Figure 1). Except for DecR, the genes for all other 12 ST-TFs were mapped into a type-A organization on the *E. coli* K-12 genome (Table 1). After continued gSELEX screening, we found 11 additional ST-TFs (CsqR, CusR, HprR, NorR, PepA, PutA, QseA, RspR, UvrY, ZraR and YqhC), of which the genes for 8 ST-TFs were located in the type-A gene organization. The details of the newly identified type-A ST-TFs are described below.

**TABLE 1:** List of single-target TFs.

TF	Alternative	Family	Gene organization	Effector	Function (Naming)	Regulatory target operon*

**List version-1 [Shimada et al. (2018b)]**						
BetI		TetR	Type-A	Choline	Betaine inhibitor	*betIbetBA/betT*
CecR	YbiH	TetR	Type-A		Cefoperazone-chloramphenicol sensitivity	*cecRybhRGFSR/rhlE*
DecR	YbaO	AsnC	Type-B		Regulator of cysteine detoxification	*cyuPA*
KdpE		OmpR	Type-A	AcP (KdpD)	K + uptake operon regulator	*kdpFABCD/kdpE*
LacI		GalR/LacI	Type-A	Allolactose	Lac operon regulator	*lacZYA/lacI*
MarR		MarR	Type-A	Salicylate	Multiple antibiotic resistance regulator	*marC/marRmarAB*
NanR		GntR	Type-A	N-Acetylneuraminate	N-Acetylneuramic acid regulator	*nanATEK/nanR*
NemR	YdhN	TetR	Type-A		Regiator of N-etyleneimidazole resistance	*rplB/rpiRalsBACE*
NimR	YeaM	AraC	Type-A		Regulator of 2-nitroimidazole resistance	*nimR/nimT*
RpiR		RpiR	Type-A	D-Allose	Ribose utilizatiion regulator	*rplR/rpiB*
TorR		OmpR	Type-A	ApC (TorS)	TMAO reductase regulator	*torR/torC*
UlaR		DeoR	Type-A		L-Ascorbate utilization regulator	*ulaR/ulaG/ulaABCDEF*
UxuR		GntR	Type-A	Galacturonate, Glucuronate	Hexuronate regulator	*gntP/uxuABuxuR*
XynR	YagI	IclR	Type-A		Regulator of xylonate catabolism	*yagEF/xynR*

**List version-2 [this report]**						

CsgR	YihW	DeoR	Type-A		Sulfoquinovose catabolism regulator	*squUTS/squVcsgR*
CusR		OmpR	Type-A	AcP (CusS)	Cu-sensing regulator	*cusRcusS/cusCFBA*
HprR	YedW	OmpR	Type-A	AcP (HprS)	Hydrogen peroxide response regulator	*hpRhprS/hiuH, cusRS/cusCFBA*
NorR	YgaA	NtrC	Type-A		No reduction detoxification regulator	*norR/norVQ*
PepA		Trigger	Type-B		Peptidaase trigger regulator	*nfeF/nfeR*
PutA		Trigger	Type-A		Proline utilization trigger regulator	*putA/putP*
QseA		LysR	Type-A	aromatic carboxylic acid	Quorum-sensing regulator A	*aaeXAB/qseA*
RspR	YdfH	GntR	Type-B		Mannonate utilization regulator	*rspAB*
UvrY		LuxR	Type-B		UV response regulator	*csrB, yihA/csrC*
ZraR		NtrC	Type-A	AcP (ZraS)	Zn resistance-associate regulator	*zraP/zraSzraR*
YqhC		AraC	Type-A		Glyoxal reductase regulator	*yqhC/yqhDdkgA*

*^∗^The single-target TF gene is shown in red. Slash shows spacers between neighouring genes.*

#### NorR (NO Reduction Detoxification Regulator)

**FIGURE 2:**
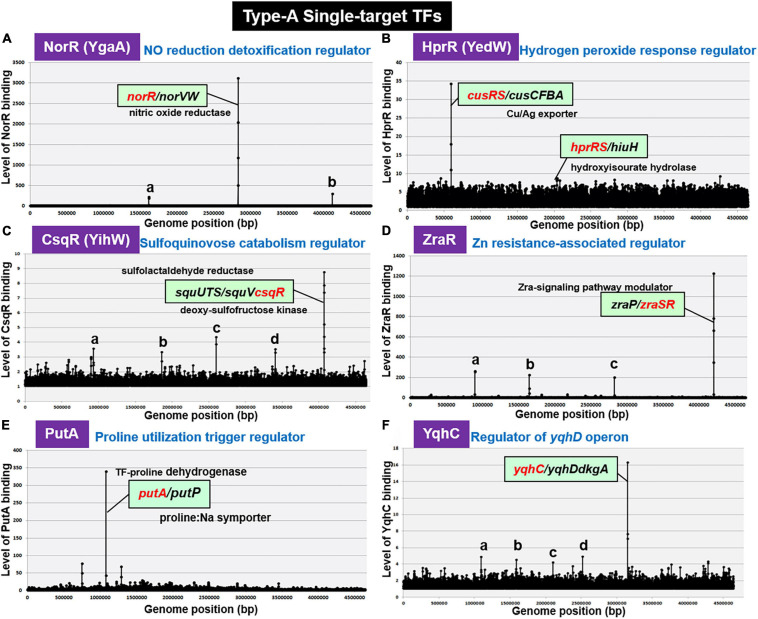
gSELEX patterns of novel type-A ST-TFs. **(A)** NorR (renamed YgaA). Two minor peaks represent: a. spacer (*sad/yneJ*); and b. spacer (*glpF/zapB*). **(B)** HprR (renamed YedW). The *hprR* gene itself forms a minor peak. **(C)** CsqR (renamed YihW). Four minor peaks represent: a. ORF (*trxB*); b. ORF (*yeaC*); c. ORF (*hyfB*); and d. ORF (*accC*). **(D)** ZraR. Three minor peaks represent: a. spacer (-/*potF*); b. spacer (*relB/ydfV*); and c. spacer (*mltB/selA*). **(E)** PutA. **(F)** YqhC. Low-level peaks with weak binding affinity to test ST-TFs disappeared after repeated gSELEX screening (for instance see Shimada et al., 2018b).

By gSELEX screening, NorR (renamed YgaA) was found to bind only to the spacer between *norR* and its target *norVW* (Figure 2A). NorR regulates the activity of both *norR* and the divergently transcribed *norVW*, which encode a nitric oxide (NO)-reducing flavorubredoxin for detoxification of NO (Lawrence, 1997; Gardner et al., 2003). This finding concurs with the bidirectional transcription of the two genes *in vivo* (Tucker et al., 2006). Under anaerobic conditions, NorR activates the transcription of the *norVW* operon (Gardner et al., 2003; Mukhopadhyay et al., 2004). A *norR* mutant is defective in anaerobic NO detoxification and is thus sensitive to reactive nitrogen intermediates. The expression of *norR* is activated in the absence of oxygen and nitrite under anaerobic conditions (Da Costa et al., 2003). Since the regulation of predicted target *norVW operon* was already established, we concluded to classify NorR as a member of ST-TFs.

#### HprR (H_2_O_2_-Response Regulator)

H_2_O_2_-response regulator (renamed YedW) forms a TCS (two-component system) with HprS (renamed YedV) response regulator. After gSELEX screening, a single high-level peak of HprR binding was detected in the spacer between the bidirectional transcription units *cusRS* and *cusCFBA* (Figure 2B), suggesting HprR as one ST-TF. CusRS is another TCS that monitors the Cu^2+^ concentration and regulates its efflux, thus indicating the cross-talk between HprRS and CusRS TCSs. Aside from this cross-talk, HprR binds, albeit at lower affinity, to the spacer between the bidirectional transcription units *hprRS* and *hiuH* for autoregulation of HprRS and for prevention of toxic compounds accumulation via removal of 5-hydroxyisourate (Hennebry et al., 2006). Interestingly, HprR recognizes the same DNA sequence as CusR but with different affinity (Urano et al., 2017). HprR and CusR regulate the same set of targets, but recognize different environmental signals: HprRS senses H_2_O_2_ levels while CusSR senses Cu^2+^ levels, thus forming a unique regulatory cascade in which the same set of genes is regulated in response to two different environmental signals via two different TCS systems. In agreement with the functional overlap between CusR and HprR, these two proteins share 51.6% similarity in amino acid sequence.

#### CsqR (Sulfoquinovose Catabolism Regulator)

Recently we identified the involvement of CsqR (renamed YihW) in repression of the genes involved in the catabolism of sulfoquinovose (SQ), a hydrolysis product of sulfoquinovosyl diacylglycerol (SQDG) (Shimada et al., 2019). After gSELEX screening, CsqR was found to bind strongly to the spacer between *squUTS* and *squTcsqR* operons (Figure 2C). SquUTS (renamed YihUTS) is involved in degradation of plant-derived sulfoquinovose (Denger et al., 2014). The activity of the CsqR transcriptional regulator is controlled by SQ and sulfoquinovosyl glycerol (SQG) during the exponential growth phase. Both SQ and SQG act as inducers for the *squUTS* operon and *squV* genes as well as for expression of *csqR* (Denger et al., 2014). We then classified CsqR as a member of type-A ST-TF group.

#### ZraR (Zn^2+^ Resistance-Associated Regulator)

Zn^2+^ resistance-associated regulator (renamed HydG), the response regulator of ZraSR TCS, controls the expression of genes involved in tolerance to high levels of Zn^2+^ (Lee et al., 2005). After six-cycles of gSELEX screening, ZraR was found to bind to a single target located inside the spacer of bidirectional transcription units *zraP* and *zraSR* (Figure 2D), indicating autoregulation of *zraSR*. In parallel, the binding specificity of ZraR to *zraP/zraSR* intergenic region was confirmed by using the gSELEX-clos (cloning-sequencing) method. Among the total of 86 independent clones, 81 clones carried the *zraP/zraSR* intergenic sequences (data not shown), indicating the highest affinity of ZraR to this zraP/zraSR intergenic spacer. The *zraP* gene, the sole regulatory target of ZraR, encodes the accessory protein of the ZraSR-signaling pathway (Rome et al., 2018). ZraP, a Zn^2+^-containing periplasmic protein with chaperone activity, leads to increased zinc tolerance (Petit-Härtiein et al., 2015). In addition to this high-affinity *zraP-zraSR* peak, several low-affinity peaks were identified (Figure 2D). Using ChIP-seq assay, however, a total of 25 additional regulatory targets were identified (Rome et al., 2018), of which the majority are involved in the envelope stress response. The low-affinity peaks are not included in the list identified by ChIP-chip. Noteworthy is that ZntR is a member of RpoN sigma-dependent NtrC family TF, which contains a central ATP-binding AAA + domain with unknown function. One possibility is possible influence of ATP on the target selection *in vitro.* Here we tentatively classified ZraR as a member of conditional ST-TF.

#### PutA (Proline Utilization Trigger Regulator)

Some enzymes, referred to trigger enzymes, acquired a DNA-binding domain and act as TF in the absence of substrates (Commichau and Stulke, 2008). *E. coli* K-12 contains five species of the trigger enzymes, BirA (biotin-protein ligase), NadR (nicotinamide mononucleotide adenyltransferase), PepA (aminopeptidase), PutA (proline dehydrogenase), and PyrH (UMP kinase), of which PepA and PutA were found members of the ST-TF group after gSELEX screening. PutA is one of the bifunctional trigger regulators that functions as a transcriptional repressor and membrane-associated proline dehydrogenase. PutA binds only to the spacer between the bidirectional transcription units *putA* itself and *putP* (Figure 2E). PutP is a Pro/Na^+^ symporter responsible for the uptake of proline (Reizer et al., 1994). In the presence of proline, PutA is associated with the cytoplasmic membrane and acts as an enzyme that catalyzes two-step reactions of the proline degradation pathway: oxidation of proline by proline dehydrogenase and subsequent oxidation to glutamate by pyrroline-5-carboxylate (P5C) dehydrogenase. In the absence of proline, PutA remains in cytoplasm and it functions as a transcriptional repressor of the *put* regulon. In the absence of proline, PutA binds to operator sequences in the *putA*-*putP* intergenic region and represses its transcription (Zhou et al., 2008).

#### YqhC (Regulator of the *yqhC-dkgA* Operon)

AraC-type YqhC has been proposed to bind and regulate the adjacent *yqhD* gene encoding glyoxal reductase (Lee et al., 2010), and its downstream *dkgA* gene encoding methylglyoxal reductase (Ko et al., 2005). These NADPH-dependent oxidoreductases are involved in detoxification of glyoxals (Lee and Park, 2017), which contain two adjacent reactive carbonyl groups, referred to as reactive electrophilic species that lead to damaging proteins and nucleic acids (Farmer and Davoine, 2007). gSELEX screening indicated only a single binding site for purified YqhC inside the spacer between *yqhC* itself and *yqhD-dkgA* (see Figure 2F). Thus, YqhC could be classified as a member of ST-TFs.

### Conditional Type-A Single-Target TFs

The regulatory function of TFs is generally controlled through structural modulation via either phosphorylation-dephosphorylation by TCS sensor kinase or interaction with effector ligands. If one of two form TFs functions as an ST-TF, we designated as a conditional ST-TF. Followings are the conditional ST-TFs so far identified.

#### CusR (Cu^2+^-Sensing Regulator)

**FIGURE 3:**
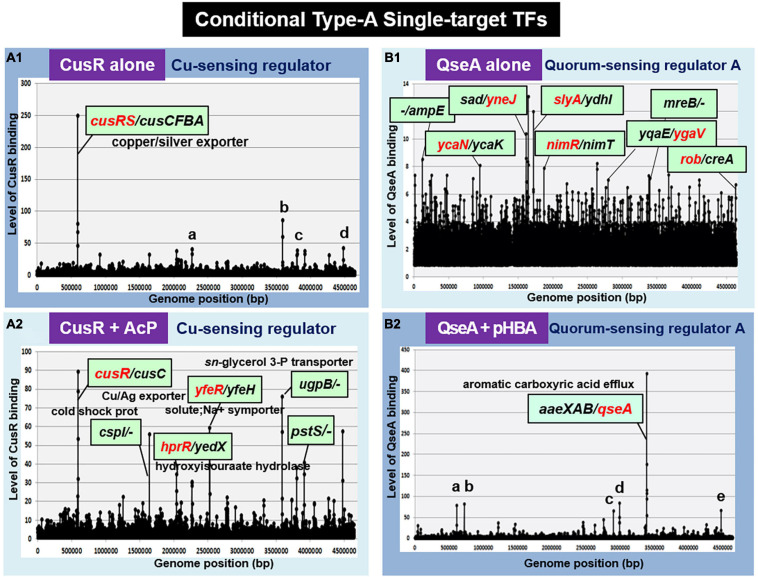
gSELEX patterns of the conditional type-A ST-TFs. **(A1)** CusR alone. One high-level peak of ST-TF was identified inside the spacer between the *cusRS* and *cuscFBA* operons. Four minor and supposedly non-specific binding peaks represent: a. ORF(*fruB*); b. spacer (*ugpB/-*); c. ORF (*rfaS*); and d. ORF (*yjgN*). **(A2)** CusR + AcP. Nine high-level peaks were identified, including the *cusR/cusC* spacer. **(B1)** QseA alone. In the absence of effector, QseA binds more than 50 sites. It remains unsolved whether these bindings represent certain physiological roles. **(B2)** QseA + pHBA. One high-level peak of ST-TF was identified. In the presence of an effector ligand pHBA, QseA recognizes and binds to a single target within the spacer between *aaeXAB* and *qseA.* Five minor and supposedly non-specific peaks represent: a. ORF (*entD*): b. spacer (-/-): c. spacer (-/-): d. spacer (-/*ygeG*): and e. ORF (*yjgL*).

Cu^2+^-sensing regulator, the response regulator of CusSR TCS, regulates the *cusCFBA* operon involved in the copper and silver efflux systems (Munson et al., 2000; Yamamoto et al., 2005) under anaerobic growth and under extreme copper stress during aerobic growth (Outten et al., 2001). In the absence of AcP, the unphosphorylated CusR was found to bind only inside the spacer between *cusRS* and *cusCFBA* operons (Figure 3A1). However, this single-target selectivity of CusR is lost in the presence of AcP (Figure 3A2). Phosphorylated CusR recognizes and binds to more than 10 targets. Thus, we classified CusR as a conditional ST-TF, which represses a single target *cusCFBA* operon in the absence of metal inducers.

In TCS signal transduction, crosstalk takes place at all three stages: recognition of external signal by the sensor kinase, phosphorylation of the response regulator by the sensor kinase, and recognition of regulatory targets by the response regulator (Yoshida et al., 2015; Yamamoto et al., 2018). Previously, we identified that CusR is phosphorylated by not only CusS, the cognate pairing sensor kinase, but also other TCS sensor kinases, including UhpB (the sensor kinase of UhpAB TCS) and HprS (the sensor kinase of HprSR TCS) (Yamamoto et al., 2005; Urano et al., 2017). Accordingly, CusR phosphorylation should take place not only in the presence of copper stress but also in the presence of H_2_O_2_ (Urano et al., 2015, 2017). In concert with this cross-regulation, phosphorylated CusR regulates the *hprRS* TCS genes (Figure 3A2). indicating the stage 2 crosstalk between CusSR and HprSR.

#### QseA (Quorum Sensing Regulator A)

**FIGURE 4:**
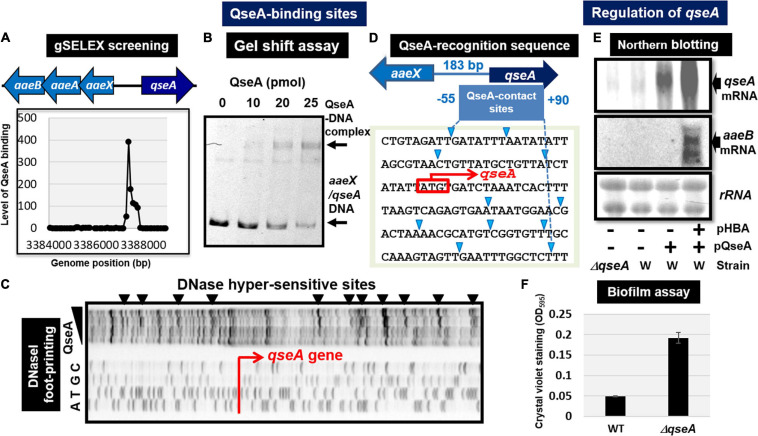
Functional analysis of QseA. **(A)** Expanded gSELEX pattern of QseA. gSELEX pattern is expanded to show the QseA-binding level near the single major peak inside the spacer between *aaeXAB* and *qseA*. **(B)** Gel shift assay of QseA. **(C)** DNase-I footprinting of the QseA-binding sequence above the *qseA* promoter region. DNase-I hypersensitive sites are indicated by triangles along the promoter sequence. **(D)** QseA recognition sequence determined by DNase-I footprinting. DNase-I hypersensitive sites are indicated by triangles (shown in blue). **(E)** Northern blot analysis of *qseA* and *aaeB* mRNAs. RNA was isolated from wild-type and *qseA*-deletion mutant *E. coli* K-12 grown in the presence and absence of pHBA. RNA was subjected to Northern blot analysis using the *qseA* and *aaeB* probes. The amounts of QseA target mRNAs were measured as relative levels of 23S and 16S rRNAs stained with methylene blue. **(F)** Biofilm assay of wild-type and *qseA*-deletion mutant using crystal violet staining method.

Quorum sensing regulator A (renamed YhcS) of the LysR family was first identified as a quorum-sensing regulator that is also involved in the expression of the pathogenicity island-located locus of enterocyte effacement (LEE) genes in pathogenic *E. coli* strains EHEC and EPEC (Sperandio et al., 2002). The *qseA* gene is present in non-pathogenic *E. coli* K-12. Thus QseA was predicted to play a role in regulation of *E. coli* K-12 genes. In fact, QseA was identified to regulate the divergently transcribed *aaeXAB* operon encoding the AaeAB efflux pump of aromatic carboxylic acids such as *p*-hydroxybenzoic acid (pHBA) (Van Dyk et al., 2004), which plays a role in alleviating the toxic effect of aromatic carboxylic acids. gSELEX screening of QseA in the presence of effector pHBA identified a single peak inside the spacer between *aaeXAB* and *qseA* (Figures 3B2, 4A), implying pHBA-bound QseA as a ST-TF. To confirm the *in vitro* binding of QseA to the *aaeX/qseA* intergenic region, we carried out a gel shift assay for the detection of QseA-*aaeX/qseA* DNA complexes. As a result, the DNA probe formed QseA-concentration-dependent QseA-DNA complexes (Figure 4B). The results of DNA foot-printing indicated that QseA binds to a wide range of 133 bp-long DNA sequences, forming at least 11 hypersensitive sites against DNase-I (Figures 4C,D). The QseA protein, which is 309 residues long, might associate cooperatively with its own promoter region.

In wild-type *E. coli* K-12, the expression of QseA was low as detected by Northern blot analysis. The expression of QseA increased after expression *in trans* of QseA using the expression plasmid pQseA (Figure 4E). In the presence of both pQseA and pHBA, the levels of both *aaeA* and *aaeB* mRNAs (the regulatory targets of QseA) increased (Figure 4E), thus supporting the positive regulatory role of QseA in pHBA-dependent expression of its single target *aaeXAB* operon. With respect to pHBA-depending *aaeXAB* activation, QseA should better be defined as AaeR (Van Dyk et al., 2004). The known physiological role of QseA in non-pathogenic *E. coli* K-12 is the efflux of aromatic carboxylic acids, indicating that pHBA-found QseA is the functional TF form. Thus, we tentatively classified QseA as a ST-TF. In the absence of effector pHBA, however, QseA binds to several sites along the *E. coli* K-12 genome (Figure 3B1), including the genes related to biofilm formation. The biofilm formation *in vivo* was induced in the absence of the *qseA* gene as detected by crystal violet staining (Figure 4F), implying the repression role of QseA of biofilm formation. In addition, QseA was suggested to regulate several TF genes, including NimR (regulator of 2-imidazole exporter), Rob (MarA/SoxS-family stress response nucleoid-associated regulator), SlyA (MarR-family stress-response regulator), YcaN, YgaV, and YneJ (Figure 3B1), which altogether might be involved in the control of *E. coli* growth under stressful conditions. In this regard, QseA might play a role in quorum sensing in the absence of pHBA. QseA might be a bifunctional TF, and then should better be classified as a conditional ST-TF. The functional form working as a ST-TF is opposite between CusR and QseA: unmodified form for CusR; and effector-bound form for QseA.

### Identification of Novel Type-B Single-Target TFs

**FIGURE 5:**
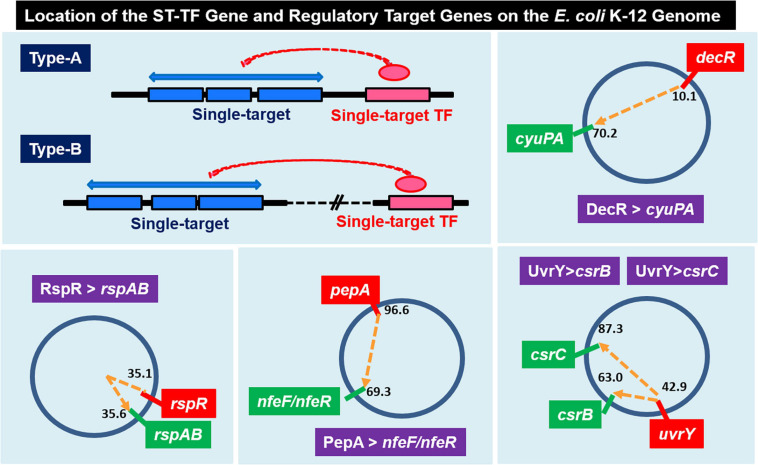
Location of the ST-TF gene and its regulatory target gene on the *E. coli* K-12 genome. [Type-A] The ST-TF gene is located adjacent to its regulatory target genes. [Type-B] The ST-TF gene is located in a separate position from its target genes. Four type-B gene sets have been identified: *decR* and its target *cyuPA* (described in version-1 list) (Shimada et al., 2018b); *rspR* and its target *rspAB*; *pepA* and its targets *nfeA*/*nfeR* genes; *uvrY* and its targets *csrB* and *csrC* genes (all described in this report). The number indicates the position of the gene on the *E. coli* K-12 genome as centisome.

Among the 13 ST-TFs listed in the first version of ST-TF list (Shimada et al., 2018b), only *decR* was not directly connected with its regulatory target genes on the *E. coli* K-12 genome (Table 1). The separated localization of a ST-TF gene and its regulatory target genes, designated as type-B gene organization (Figure 5), is rare in a group of ST-TFs. In addition to DecR, we identified here three other type-B single-target TFs, including PepA, RspA, and UvrY (Table 1). The details of type-B ST-TFs are described as follows.

#### PepA (Peptidase Trigger Regulator)

**FIGURE 6:**
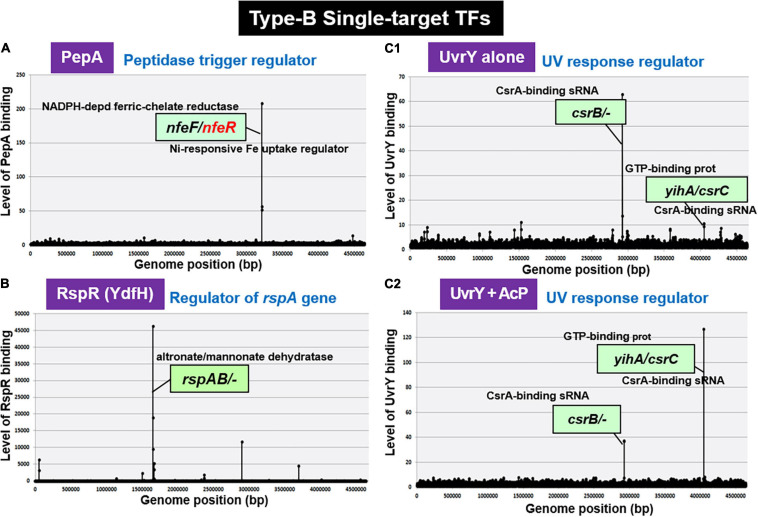
gSELEX patterns of novel type-B ST-TFs. The ST-TF gene and its regulatory target gene(s) are located separately on the *E. coli* K-12 genome. **(A)** PepA binds to a single target inside the spacer between *nfeF*, which encodes a NADP-dependent ferric-chelate reductase, and *nfeR*, which encodes a Ni-responsive Fe^3+^ uptake regulator. **(B)** RspR binds to a single target upstream of the *rspAB*, which encodes a predicted Zn^2+^-dependent D-mannose dehydrogenase. **(C1,C2)** UvrY recognizes and binds to the region upstream of *csrB* and the spacer region of *yihA/csrC*, but the binding affinity may shift depending on level of phosphorylation.

*Escherichia coli* K-12 W3350 contains five species of the enzyme-TF fusion trigger, of which BirR and NadR belong to the group of multi-target TFs (Shimada, T. and Ishihama, A., unpublished) while another trigger PutA (proline dehydrogenase) is a type-A ST-TF (see Figure 2E). In contrast, trigger PepA (aminopeptidase A/I) is a type-B ST-TF (see Figure 5). PepA peptidase is known to bind DNA and control transcription of some other genes (Devroede et al., 2004). After six-cycle gSELEX screening in the absence of other regulatory proteins, PepA was found to bind only to the spacer between *nfeF*, which encodes NADPH-dependent ferric reductase, and *nfeR*, which encodes a Ni-responsive Fe^3+^ uptake regulator (Figure 6A). This finding indicates the participation of PepA in the regulation of uptake and utilization of ferric ions. Previously, however, PepA was proposed to bind to several DNA regions, including the regulatory region upstream of the *carA* promoter (Charlier et al., 1995), implying the involvement of PepA in regulation of the *carAB* gene that plays a role in pyrimidine synthesis. Transcriptional regulation of the *carAB* promoters requires additional regulatory proteins other than PepA (Charlier et al., 2000). At least eight TFs including ArgR, ArcC, Fis, IHF, PepA, PurR, PyrH, and RutR have been proposed to be involved in this regulation (Kholti et al., 1998; Minh et al., 2009). One possibility of the failure of *carAB* promoter might be loss of the *carAB* promoter DNA after repeated cycles of gSELEX. PepA might interact with other TFs for strong binding to the *carAB* promoter (Minh et al., 2016). In the multi-factor promoters, not only the competition between TFs but also the collaboration for enhancement of DNA binding take place, which share the same binding regions near a single promoter (Ogasawara et al., 2010). It also remains unsolved how the enzyme (aminopeptidase in PepA) of trigger TFs influence the TF activity. Further studies are needed for understanding the selectivity control of regulatory targets by PepA.

#### RspR (Regulator of Starvation-Sensing *rspAB*)

**FIGURE 7:**
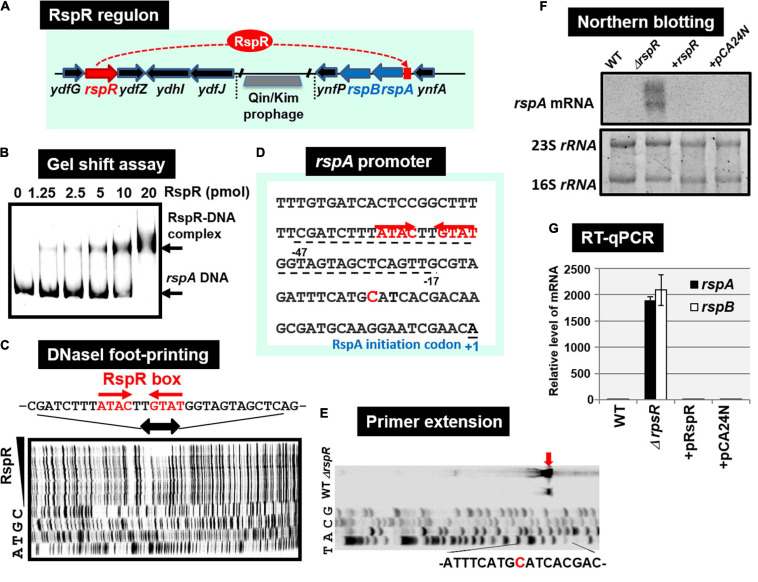
Functional analysis of RspR. **(A)** Gene organization of the RspR regulon. The *rspR* gene and its regulatory target *rspAB* operon are separated by Qin/kim prophage. **(B)** Gel shift assay of RspR. **(C)** DNase-I footprinting of the RspR-binding sequence around the *rspA* promoter region. RspR box is indicated by red arrows. **(D)** The *rspA* promoter sequence is shown with the *rspA* transcription start site (indicated by blue T) and the translation initiation site (indicated as + 1). The sequence protected by RspR is shown with dotted line below the *rspA* promoter sequence. **(E)** Mapping the *rspA* promoter via primer extension. The transcription start site is indicated by blue arrow, and the start nucleotide A on the *rspA* mRNA is shown in red. **(F)** Northern blot analysis of *rspAB* transcript. **(G)** RT-qPCR analysis of *rspAB* transcript. In both F and G experiments, RNA was isolated from wild-type, the *rspA* mutant, the mutant with RspR over-expressing plasmid pRspR, and the mutant with empty vector pCA24N.

Using the gSELEX screening system, we identified regulatory functions for approximately 20 uncharacterized TFs. Among the 20 as yet uncharacterized TFs, YdfH of the GntR-family exhibited one major peak in the gSELEX pattern after six cycles of gSELEX screening (Figure 6B). The binding site of YdfH is located upstream of the *rspAB* operon but downstream of the *ynfA* gene, which encodes an inner membrane protein, indicating that *rspAB* is the sole target of YdfH. We predicted the *rspAB* operon as the single target of YdfH and then renamed YdfH to RspR, a member of ST-TF group. Overexpression of *rspAB* interferes with the synthesis of stationary phase-specific RpoS sigma, thereby leading to the name *rsp* (regulatory-in-stationary-phase) genes (Huisman and Kolter, 1994). However, RspAB is supposed to be Zn^2+^-dependent D-mannose dehydrogenase (Gerlt et al., 2005). Functional connections, however, remain unsolved. In the *E. coli* K-12 genome, the *rspR* gene and its target *rspAB* operon are separated by a 26,103 bp-long insertion including the Qin/Kim prophage (Figure 7A), and thus the *rspR* gene is classified as a type-B ST-TF organization. In some *E. coli* family bacteria, however, the Qin/Kim prophage is not inserted in this spacer, supporting the predicted evolution of this TF and target organization. To confirm the *in vitro* binding of RspR to the *rspA* promoter region, we carried out a gel shift assay for the detection of RspR-*rspA* DNA complexes. As a result, the DNA probe formed RspR-concentration-dependent RspR-DNA complexes (Figure 7B). Using DNase-I footprinting assay, a 31 bp long sequence was protected (Figure 7C), which included the ATACnnGTAT palindromic sequence, referred to as the RspR-box, in the center (Figure 7D). To understand the regulation mechanism, a primer extension assay was performed for the *rspA* promoter region to identify the transcription start site. Total RNA was purified from wild-type and *rspR*-defective mutant strains and subjected to the assay. As a result, clear signals were detected at the position of the C base, which is located 30 bp upstream from the RspR ATG initiation codon (Figure 7E). The intensity of the signal that was detected was higher in the *rspR* mutant than in the wild-type strain, indicating that RspR represses the *rspA* promoter. This is in good agreement with typical repressor, which inhibits binding of RNA polymerase to the promoter via overlapping the RNAP and RspR binding sites. In order to experimentally confirm the regulation of *rspAB* by RspR, we next performed northern blot analysis using a DIG-labeled *rspA* probe for wild-type, *rspR*-defective mutant, and RspR overexpressing strains together with wild-type strains carrying the empty-vector. Total RNA was purified from each strain in both exponential and stationary phases. In the log phase, an approximately 2.3 kb signal corresponding to the size of the *rspAB* operon was observed only in the genome of *rspR* mutant strain (Figure 7F). The results obtained by northern blot analysis were also confirmed through RT-qPCR using probes for both *rspA* and *rspB*. The results of both northern blot and RT-qPCR analyses indicated an increase in the mRNA levels of *rspA* and *rspB* in the absence of RspR (Figure 7G). The expression levels of *rspA* and *rspB* in the stationary phase were essentially the same as those in the log phase (data not shown). Taken together, we concluded that RspR represses *rspA* during both log and stationary phases.

#### UvrY (UV Response Regulator)

After gSELEX screening, UvrY alone exhibited one major peak at upstream of the *csrB* gene encoding sRNA CsrB, but downstream of the *cyd* gene (Figure 6C1), indicating the *csrB* gene as the major target of unphosphorylated UvrY. In addition, a low-level peak was detected within the spacer between the *yihA* gene encoding a GTP-binding protein and the *csrC* gene encoding another sRNA CsrC. These two peaks were also identified during phosphorylation of UvrY in the presence of AcP; however, the peak of *yihA/csrC* was higher than the peak of *csrB* (Figure 6C2). The selectivity of regulatory targets of UvrY was found to change depending on the concentration of effector AcP, thereby the level of protein phosphorylation. As in the case of other phosphorylation-dependent control of TF activities, the target selectivity of UvrY should change depending on the level of protein modification. Our gSELEX screening results of UvrY agree well with the proposed regulatory function of BarA/UvrY TCS in central carbon metabolism via regulation of the small non-coding RNAs, such as CsrB and CsrC (Zere et al., 2015). Both *csrB* and *csrC* sRNAs bind to the RNA-binding protein CsrA (carbon storage regulator) to remove it from its target mRNAs (Romeo et al., 2013), thereby allowing the translation of a set of mRNAs under the repression by CsrB and CsrB sRNA (Romeo et al., 2013). In concert with this prediction, the expression of CsrB and CsrC is also under different controls, involving regulators other than UvrY, such as ppGpp (Edwards et al., 2011), CRP (Pannuri et al., 2016), and IHF (Romeo and Babitzke, 2018).

## Discussion

### Classification of TFs Based on the Number of Regulatory Targets

The model prokaryote *E. coli* K-12 contains approximately 300 species of the DNA-binding TFs, of which regulatory targets have been identified mainly based on *in vivo* analyses using varieties of modern molecular genetic approaches (see Introduction). The majority of regulatory targets thus identified, however, represent those indirectly affected in the absence of TF gene or over-expression of test TF (Ishihama et al., 2016; Shimada et al., 2018a). We then switched to employ *in vitro* approaches such as gSELEX (Shimada et al., 2005, 2018a) and PS-TF screenings (Shimada et al., 2014c; Yoshida et al., 2018) using the collection of purified TFs and a library of genome DNA segments from a single and the same *E. coli* K-12 strain. Sequences of the protein-bound SELEX DNA fragments was previously determined by cloning and sequencing (gSELEX-clos), but recently determined by using tilling array (gSELEX-chip) to increase the resolution (Shimada et al., 2018a) (for details see section “Materials and Methods”). The resolution could be amplified by determination of TF-binding sequence with use of foot-printing techniques or DNA-Seq methods. Based on the number of regulatory targets included only in this data collection but avoiding the use of public *E. coli* TF databases, we classified TFs into four groups: ST-TFs, local TFs, global TFs, and nucleoid-associated TFs in the increasing order (see Figure 1). At present, the apparently clear boundary exists between these four groups but once gSELEX data are established for all *E. coli* K-12 TFs, but we will propose an improved classification once we get the whole set of regulatory targets for more TFs from the same *E. coli* K-12. For instance, gSELEX data have not been established for some proposed global regulators such as Fnr and NarL (Martınez-Antonio and Collado-Videsy, 2003; Browning and Busby, 2016). In the coming new classification system, the boundary between the current four TF groups could be modified into more than four groups.

### Gene Organization of ST-TFs on the *E. coli* Genome

**FIGURE 8:**
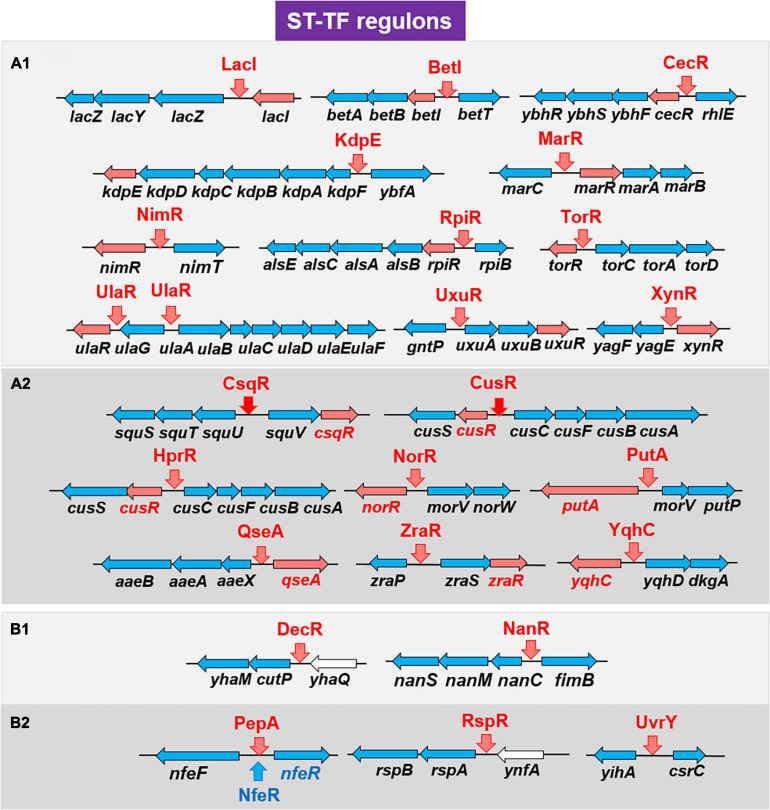
Detailed organization of the ST-TF gene and the regulatory target gene(s). A total of 24 ST-TFs from the previous report (Shimada et al., 2018a) **(A1,B1)** and this report **(A2,B2)** were classified. **(A)** Type-A organization. The ST-TF proteins and their regulatory target genes are shown in red. **(B)** Type-B organization. The ST-TF proteins, shown in red, regulate separately located target genes. For details see text.

Overall, a total of 24 ST-TFs have been identified and have been included, 13 ST-TFs in Shimada et al. (2018b) and 11 ST-TFs in this report. Most of these ST-TF genes are organized in the type-A genetic system, in which the TF genes are located close or adjacent to their regulatory target genes (Figure 8). The type-A gene set can be easily transferred into *E. coli* K-12 from other bacteria existing in the same environment in nature. Moreover, the type-A gene set can be easily retained in *E. coli* if the products of target genes confer a benefit to *E. coli*. One major pathway of gene transmission is phage infection. Phage fossils of 10 prophages exist in *E. coli* K-12 (Casjens, 2003; Wang et al., 2010), which together comprise approximately 3.6% of the *E. coli* K12 genome and include 14 TF genes (Yamamoto et al., 2018). Inside these prophage regions, at least 10 TF genes can be detected, including AlpA, AppY, CroE, DicA, DicC, PerR, XynR, YbcM, YfjR, and YmfK (Table 2). Except for AppY, the regulatory targets of other TFs can be detected within the prophage regions, keeping the type-A gene organization. In case of AppY (acid phosphatase regulator), the regulatory target genes *appCBX*, which encodes cytochrome *bd*-II oxidase, and *hyaABCDEF*, which encodes the hydrogenase, are located outside the prophage regions in the *E. coli* K-12 genome (Atlung et al., 1997; Giuffrè et al., 2012). The shift in the gene organization of ST-TF and its target genes from type-A to type-B may be related to the cross-talk between host *E. coli* and prophages. After prolonged coexisting life cycles, prophage-encoded TFs might get chances to regulate some host genes. Likewise, *E. coli* TFs might start to control prophage target genes. Using gSELEX and PS-TF screening systems *in vitro* could be used toward understanding the cross-communication. Along this line, the communication of host *E. coli* TFs and pathogenic island TFs in pathogenic *E. coli* could also be another hot spot of the coming age of TF research.

**TABLE 2:** TF genes inside prophages in the *E. coli* K-12 genome.

TF	Prophage	Length (bp)	Regulation targets	Location
AlpA	CP4-57	27,332	*intA* operon	CP4-57 prophage
AppY	DLP12	18,849	*hyaABCDEF,appCBA*	Hose core genome
CroE	e14	15,193	*croE* operon	e14 prophage
DicC	Qin	19,752	*dicB* operon	Qin prophage
DicA	Qin	19,752	*dicB* operon	Qin prophage
PerR	CP4-6	27,332	*perR* operon	CP4-6 prophage
XynR	CP4-6	27,332	*yagA* and *agEF* operons (xylose catabolism)	CP4-6 prophage
YbcM	CLP12	18,849	*ybcM* operon (stress response)	CLP12 prophage
YfjR	CP4-57	22,030	*yfjR* operon (biofilm formation)	CP4-57 prophage
YmfK	e14	15,193	SOS-sensitive repressor	e14 prophage

## Conclusion

Using the *in vitro* gSELEX screening system with use of purified TFs and a collection of genome DNA segments, we have identified the whole set of regulatory targets for about half of the total of approximately 300 species of TF from the model prokaryote *Escherichia coli* K-12 W3110. Based on the number of regulatory targets, TFs could be classified into four groups in increasing order: single-target regulator (ST-TF); local regulators; global regulators; and nucleoid-associated regulator. A total of 11 ST-TFs were newly identified, constituting together with 13 hitherto identified ST-TFs (including in version-1 list) the minority group of *E. coli* K-12 TFs. On the basis of organization of ST-TF gene and its target gene(s) on the *E. coli* K-12 genome, these 24 ST-TFs were classified into adjacently arranged type-A (20 species) and separated type-B (4 species) organization. The origin and evolution of ST-TFs are discussed.

## Data Availability Statement

The datasets presented in this study can be found in online repositories. The names of the repository/repositories and accession number(s) can be found below: https://shigen.nig.ac.jp/ecoli/tec/download/, gSELEX data is reposited in TEC database (https://shigen.nig.ac.jp/ecoli/tec/tfmap/#), Nucleic Acids Research (https://academic.oup.com/nar/article/44/5/2058/2465256), and all of the gSELEX data in this manuscript are available in the database.

## Author Contributions

TS, HO, IK, and NK performed the experiments. TS and HO conducted the gSELEX screening. AI conducted and designed the project. AI and TS wrote and edited the manuscripts. All authors read and approved the manuscript.

## Conflict of Interest

The authors declare that the research was conducted in the absence of any commercial or financial relationships that could be construed as a potential conflict of interest.

## Footnotes

1 www.shigen.nig.ac.jp/ecoli/tec/

## Supplementary Material

Supplementary Table 1 Primers used in this study.
